# DNA Barcode Sequence Identification Incorporating Taxonomic Hierarchy and within Taxon Variability

**DOI:** 10.1371/journal.pone.0020552

**Published:** 2011-08-16

**Authors:** Damon P. Little

**Affiliations:** Lewis B. and Dorothy Cullman Program for Molecular Systematics, The New York Botanical Garden, Bronx, New York, United States of America; American Museum of Natural History, United States of America

## Abstract

For DNA barcoding to succeed as a scientific endeavor an accurate and expeditious query sequence identification method is needed. Although a global multiple–sequence alignment can be generated for some barcoding markers (e.g. *COI*, *rbcL*), not all barcoding markers are as structurally conserved (e.g. *matK*). Thus, algorithms that depend on global multiple–sequence alignments are not universally applicable. Some sequence identification methods that use local pairwise alignments (e.g. BLAST) are unable to accurately differentiate between highly similar sequences and are not designed to cope with hierarchic phylogenetic relationships or within taxon variability. Here, I present a novel alignment–free sequence identification algorithm–BRONX–that accounts for observed within taxon variability and hierarchic relationships among taxa. BRONX identifies short variable segments and corresponding invariant flanking regions in reference sequences. These flanking regions are used to score variable regions in the query sequence without the production of a global multiple–sequence alignment. By incorporating observed within taxon variability into the scoring procedure, misidentifications arising from shared alleles/haplotypes are minimized. An explicit treatment of more inclusive terminals allows for separate identifications to be made for each taxonomic level and/or for user–defined terminals. BRONX performs better than all other methods when there is imperfect overlap between query and reference sequences (e.g. mini–barcode queries against a full–length barcode database). BRONX consistently produced better identifications at the genus–level for all query types.

## Introduction

The goal of DNA barcoding is to identify biological specimens using a short (ca. 650 bp) standardized region of DNA in a manner analogous to the use of Universal Product Codes to identify consumer goods [1]–[5]. Without an accurate and expeditious query sequence identification method, barcoding is restricted to the gathering of reference sequences. Building such a database is laudable, but of limited practical application if query sequences cannot be accurately identified.

Consumers of DNA barcodes are interested in placing their query sequences within the taxonomic hierarchy (i.e. classifying a specimen). Conventional Sequence IDentification Engines (SIDEs) such as FASTA [6] or BLAST [7] can be used for DNA barcode identification, but implementations of sequence similarity methods are often ‘corrected’ to overcome biological (e.g. mutation) or sampling bias. These ‘corrections’ may unintentionally obscure the minuscule sequence variation among closely related species.

In addition, conventional SIDEs assume that the reference sequence(s) that is (are) most similar to the query sequence is (are) the best estimate of query identification. Although this may be true from the standpoint of overall sequence similarity, classifications are most efficient when they use character–based special similarity (i.e. shared similarity due to common ancestry) rather than overall similarity [8], [9]. Character–based special similarity can either be used directly–in the form of phylogenetic trees–or implicitly–in the form of hierarchic taxonomic descriptors. To date, SIDEs that use evolutionary information are primarily adaptations of more conventional character–based phylogenetic methods [10]–[13].

SIDEs based upon phylogenetic methods face two major obstacles: First, tree–search is an NP–hard problem–with the number of possible solutions becoming impossibly large with even a small number of terminals [14]. Although a variety of efficient search heuristics are available [15]–[19], it is not computationally practical to analyze more than a few thousand terminals with current hardware. For DNA barcoding, various shortcuts have been proposed to either limit the size of the reference database and/or limit the tree–search [11], [20], [21]. Second, character–based phylogenetic methods require a multiple–sequence alignment. The contradictory requirements for a barcoding marker to be hypervariable–in order to distinguish among closely related species–yet simultaneously be highly conserved–to allow for ‘universal’ PCR primers–results in the selection of markers fraught with alignment difficulties. The impact of alignment on phylogenetic accuracy and in turn sequence identification is great [11], [22], [23]. An algorithm designed to overcome alignment ambiguity while simultaneously using phylogenetic information has been proposed, but ATIM [11] is so time inefficient that it is not useful in practice.

In addition to finding the best matching reference sequence, DNA barcoding SIDEs must confront within taxon variability [11], [21], [24]. Although it is not possible to unambiguously classify a specimen using a single barcode marker in the presence of variation shared among taxa (due to either ancestral polymorphism and/or introgression), conventional SIDEs may output an unambiguous identification simply as a result of artificial variation in sequence length.

### The BRONX algorithm

BRONX (Barcode Recognition Obtained with Nucleotide eXposés) is a novel SIDE designed to use an uncorrected character–based measure of similarity, work with difficult to align markers, capitalize upon knowledge of hierarchic evolutionary relationships, indicate ambiguous classification assignments, and account for within taxon variation.

**Figure 1:**
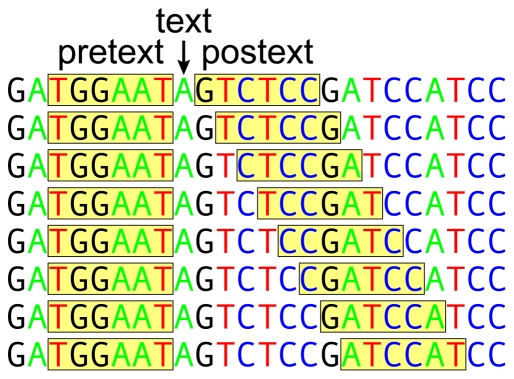
An example of context (pretext/postext) and text extraction. The size of the pretext/postext used, and the range of text sizes stored, may vary by implementation.

BRONX reduces reference sequences to a series of characters defined by flanking context (‘pretext’ and ‘postext’; Fig. 1) thereby avoiding alignment difficulties. Named terminals, be they species, higher–level taxa, or unnatural terminals of interest (e.g. pathogens) are reduced to exhaustive composite exposés. For each terminal, the exposé consists of a list of all observed sequence fragments (text) and their flanking context. This minimizes misidentifications arising from shared alleles/haplotypes and allows for the placement of undescribed (or unsampled) species within higher–level terminals. BRONX identifies queries as named terminals by first matching the context of the query sequence to the context of the reference exposés. Where there is matching context, the similarity between the query and the reference can be calculated (see Methods for additional details). BRONX in effect mimics the procedures used in traditional morphological systematics–each composite exposé is equivalent to a taxonomic morphological description where some characteristics provide context for others (e.g. hair on the midvein of leaves).

This paper aims to test the accuracy of BRONX sequence identification against leading published SIDEs. Publicly available data for the core plant barcode markers (*matK* and *rbcL*; [25]) was used in preference to animal barcode data (*CO1*; [5]) because the plant two marker system represents a more rigorous challenge to SIDE performance–one that has largely been ignored by the designers of SIDEs up until this point.

## Methods

### Barcode data

A dataset of plant core barcode markers–*matK* and *rbcL*–was extracted from publicly available sources. Sequences were included only if both markers were obtained from the same individual. Sampling was limited to digitized literature available to the author in which the relationship between sequence accessions and vouchers was explicit. The taxonomy of the original publication was used for all analyses. The resulting dataset included portions of complete plastid genomes and sequences generated for phylogenetic, biogeographic, and barcoding studies [26]–[84]. For each marker, a global multiple–sequence alignment was calculated and refined with MUSCLE 3.7 [85]. Sequences were trimmed, using the multiple–sequence alignment, to include only sequence that would be amplified if primers *matK* 3F (5′-CGT-ACA-GTA-CTT-TTG-TGT-TTA-CG-AG-3′) and 1R (5′-ACC-CAG-TCC-ATC-TGG-AAA-TCT-TGG-TTC; Ki–Joong Kim, Korea University, pers. comm) or *rbcL* aF (5′-ATG-TCA-CCA-CAA-ACA-GAG-ACT-AAA-GC-3′ [49]) and aR (5′-GAA-ACG-GTC-TCT-CCA-ACG-CAT-3′ [34]) had been used. Leading and trailing ‘N’ codes were deleted. The final dataset had 2083 sequences of each marker representing 990 genera and 1745 species (Dataset S1).

### Severity of identification tests

For a SIDE to succeed at the species– or genus–level, all sequences for a given species had to be correctly identified to the exclusion of sequences from other species or genera, respectively (i.e. ambiguous identifications were considered incorrect). The percentage of queries resulting in correct identifications is equivalent to sensitivity [i.e. true positives/(true positives+false negatives)]. Tests of species–level identification were classified either as ‘weak’ tests–those for which no congener is represented in the dataset (*n* = 784)–or as ‘strong’ tests–those for which congeners are included (*n* = 961). Reference datasets included 1745 sequences for each marker (one per species). If a species was represented by more than one individual in the full dataset, the individual with the highest *matK* ‘length/completeness score’ [11] was retained in the restricted dataset (ties were arbitrarily resolved). All 2083 sequences were used for queries.

### Mini–barcodes

To test SIDE performance using mini–barcode [86] data, each of the 2083 query sequences was reduced to a single short segment–the size (100–200 bases) and the position of the segment was randomly chosen within each query sequence. The mini–barcode was queried against a full–length reference database as described above. The original mini–barcode proposal [86] called for the use a single highly–informative segment, but such a segment has not yet been identified in plants. Currently, researchers try many combinations of primers on poor quality DNA extracts and eventually sequence a small, arbitrary positioned, fragment that varies from species to species. The location of such fragments are not random per se, but simulating the interaction between taxon specific sequence variation, the degradation of DNA, the PCR skills of a hypothetical researcher, and the available primer complement in such a researcher's laboratory is beyond the scope of this paper. Therefore a random approach was used to mimic the current patchy recovery of sequence data from specimens with poorly preserved DNA.

### Interpretation

For each SIDE and class of identification test, the binomial distribution was used to compute confidence intervals around the observed success rate [87]–[89]. Each reference species was considered an independent test.

Tukey–type multiple comparisons tests were conducted on each class of test by summing performance across markers and tests. Full–length and mini–barcode queries were considered separately and combined. Data were arcsin transformed following [90] eq. 13.8. Tests used 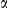 = 0.05 and followed the procedure of [90] section 24.14.

Similarity among SIDEs was quantified using Fleiss' [91]–[93] index of interrater agreement (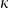). Each dataset and query type was analyzed separately.

### Simple pairwise matching

Calculations of ‘barcode gap’ magnitude (the difference between intra– and inter–specific distances) are highly sample dependent [94]. Therefore the algorithm used here only depended upon the presence or absence of a barcode gap (i.e. interspecific distance greater than zero)–a calculation that is not nearly as sample dependent. A more conservative approach, such as requiring that the minimum inter–specific distance be larger than the maximum intra–specific distance [25], was not feasible given the poor intra–specific sampling in the datasets.

#### Global alignment (Analysis 1)

The pairwise matching algorithm used here follows that of [25]: (1) All possible global (Needleman–Wunsch; [95]) pairwise alignments were calculated with MUSCLE 3.7 [85]. (2) For each pair, uncorrected p-distance was calculated using unambiguous sequence differences only. Postulated insertion/deletion (indel) events were treated as missing data. (3) As appropriate, markers were combined by summing the components of the distance measure. (4) A species was considered distinct if all inter–specific p-distances were greater than zero (contra [25]). For the mini–barcode analysis, each truncated query was aligned to all full–length reference sequences and analyzed as described above.

#### Local alignment (Analysis 2)

Steps 1–4 of Analysis 1 were followed except water 6.1.0-5 [96] was used to calculate all possible local (Smith–Waterman; [97]) pairwise alignments. The analysis was not conducted using combined queries for the mini–barcode dataset.

### Tree–based identification

#### *De novo* parsimony tree search (Analysis 3)

(1) For each marker, a global multiple–sequence alignment was calculated and refined with MUSCLE (Datastes S2 and S3). (2) Sequences for each query species were aligned to one another. They were then aligned to the reference alignment using the ‘-profile’ option of MUSCLE. (3) Postulated indels were treated as missing data, but included in the analysis using ‘simple indel coding’ [98]. (4) As appropriate, markers were combined by concatenation. (5) A fast tree search was conducted with TNT 1.1 [99] using one random addition sequence (system time was used for a random seed) and SPR branch swapping holding a single tree (‘rs0; col3; mu = rep1ho1spr;’). Ambiguously supported nodes were collapsed. *Physcomitrella patens* (Hedw.) Bruch & Schimp. was used to root all searches. (6) The least inclusive clade containing all of the query sequences was taken as the identification [11].

#### Forced parsimony tree search (Analysis 4)

Forced (constrained) parsimony tree search using a reference multiple–sequence alignment from Analysis 3 and a reference tree: (1) Reference most parsimonious trees were obtained via at least 300 ratchet tree–searches in TNT. The system time was used as the random seed and ambiguously supported branches were collapsed. For each ratchet, a single random addition sequence was swapped–exhaustively first with SPR then TBR holding up to two trees. Each of the 200 ratchet iterations was randomly re–weighted for either 8% or 10% of the informative characters and TBR swapped holding up to two trees (‘rs0; col3; ho201; rat:iter200up4do4; mu = rep100ho2rat;’). (2–5) The same as steps 1–4 of Analysis 3. (6) The tree search in step 5 Analysis 3 was used except the strict consensus of the most parsimonious trees was used as a positive constraint. (7) The resulting tree was evaluated as step 6 Analysis 3.

#### CAOS (Analysis 5)

The Characteristic Attributes Organization System (CAOS) algorithm [21], [100], [101] was compared to *de novo* and forced parsimony tree searches. (1) The reference consensus used in Analysis 4 was used to construct the CAOS rule set. Indel characters were removed from the matrix prior to rule extraction. (2) As appropriate, markers were combined by concatenation. (3) CAOS used NCBI-BLAST 2.2.13 [102] for query sequence alignment. (CAOS was not used for the mini–barcode analysis.).

#### SAP NJ (Analysis 6)

The ConstrainedNJ algorithm from the Statistical Assignment Package (SAP; [13], [20]) was used to identify query sequences. (1) A local BLAST database was searched with ‘blastall’ 2.2.17 [102]. Taxonomic annotation consisted only of genus and species names. (2) ClustalW2 (2.0.12; [103]) was used to align up to 50 sequences returned by the BLAST search (SAP was requested to return sequences from at least three genera). (3) As appropriate, markers were combined by concatenation. (4) Genus– and species–level assignments used a minimum posterior probability of 95%. Query sequences for which BLAST was unable to find any significant matches at 1.00e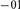 (the SAP default) were excluded from the success/failure counts. SAP could not be used for the combined marker mini–barcode analysis because BLAST could not effectively search the concatenated reference database with concatenate non–adjacent mini–barcode sequences.

#### SAP BA (Analysis 7)

The Barcoder algorithm (‘a Bayesian approach very much like MrBayes’) from the SAP [13] was used for query assignment following the steps 1–4 of Analysis 6. As described above, SAP could not be used for the combined marker mini–barcode analysis.

### DNA–BAR/degenbar (Analysis 8)

(1) Up to ten redundant distinguishing oligo nucleotide (length 10–25) sequences were located in reference sequences and their reverse complements (separated by 25 ‘N’ codes). DEGENBAR [104], [105] was given the following parameters to pick oligos: GC content 0–100%, annealing temperature 0–100

C, salt concentration 50 nM, DNA concentration 50 nM, and a maximum common substring weight of 50. For mini–barcode analysis, output from two DEGENBAR runs were used: one returned up to 10 redundant distinguishing oligos while the other returned up to 30. (2) As appropriate, markers were combined by concatenation with 25 ‘N’ codes between each marker. (3) A PERL script (http://www.nybg.org/files/scientists/degenbar.html) was used to identify query sequences using the DEGENBAR output [11].

### BLAST

#### WU-BLAST (Analysis 9)

The BLAST algorithm [7], [106] as implemented in WU-BLAST 2.0MP (2006 May 4) [107] was used to identify sequences: (1) A unified database was constructed from *matK* and *rbcL* sequences. (2) For each species, sequences were queried against the database with nucleotide–to–nucleotide comparisons using the default settings (‘blastn’). Up to 200 of the best hits were returned per query sequence (‘-B 200’). (3) As appropriate, sequences of either or both markers were used for queries. (4) The mean raw alignment score was calculated for each species using the values returned for all queries. The highest mean raw alignment score was taken to be the identification.

#### NCBI-BLAST (Analysis 10)

The NCBI implementation of the BLAST algorithm was also used (the ‘blastn’ program of blastall 2.2.17 [102]) following steps 1–4 of Analysis 9.

### BRONX (Analysis 11)

The BRONX algorithm was implemented in two PERL scripts released under GNU GPL version 2 (http://www.nybg.org/files/scientists/dlittle/BRONX.html). MySQL 5.0.67 [108] was used as a back–end database. (1) A unified database was constructed from *matK* and *rbcL* sequences using ‘BRONXpopulate.pl’. No context combinations that included IUPAC ambiguity codes were stored. (2) As appropriate, markers were combined by concatenation with 15 ‘N’ codes between each marker. (3) Query sequences were identified using ‘BRONXid.pl’.

The reference database was constructed using the following algorithm:

1. For each possible position (*p*) in a given reference sequence extract:
  - *n* contiguous pretext nucleotides [*p*, *p*+*n*] (in this implementation *n* = 6)
  - followed by *x* contiguous text nucleotides with *x* incremented from 1 to *y* [*p*+*n*+1], [*p*+*n*+1, *p*+*n*+2], … , [*p*+*n*+1, *p*+*n*+*y*] (in this implementation *y* = 8)
  - immediately followed by *n* contiguous postext nucleotides [*p*+*n*+*x*+1, *p*+*n*+*x*+1+*n*].
2. For each reference sequence, store all pretext/text/postext combinations.
3. For each terminal, create a composite exposé of all pretext/text/postext combinations known for the terminal.

The reference database was queried using the following algorithm:

1. For each possible position in the query sequence and its reverse complement, extract context and text as described above, but with *x* fixed rather than incremented (in this implementation *x* = 3).
2. If the pretext/postext combination is found among the reference exposés, score each reference terminal for the combination that is shared with the query sequence (see below).
3. If the pretext/postext combination does not match a combination in the reference exposés:
  - extract all postext combinations from the reference exposé that follow the current pretext
  - determine which of the known postext sequences is physically nearest to the current pretext
  - score each terminal in the reference exposé using the nearest pretext/postext combination (see below)
4. The reference terminal(s) with the highest final score is(are) considered the identification.

A variety of scoring functions are possible. The simplest function increments a terminal's score by one for each matching pretext/text/postext combination. Thus, the final score for each terminal can vary between zero and the query sequence length with zero awarded complete mismatches and sequence length awarded to exact matches. Several other scoring functions were used on an experimental basis (e.g. differential scoring of text versus pretext/postext), but did not appear to improve identification success (data not shown).

## Results and Discussion

The use of GenBank data necessitates an assumption of underlying data quality that cannot be independently verified without great difficulty. As a result, I assumed that there were no sequencing errors, that all specimens were consistently identified, and that the taxonomy used was sound. Given these assumptions, the results presented here allow one to choose the most accurate SIDE(s) for barcode data analysis.

### Severity of identification tests

**Figure 2:**
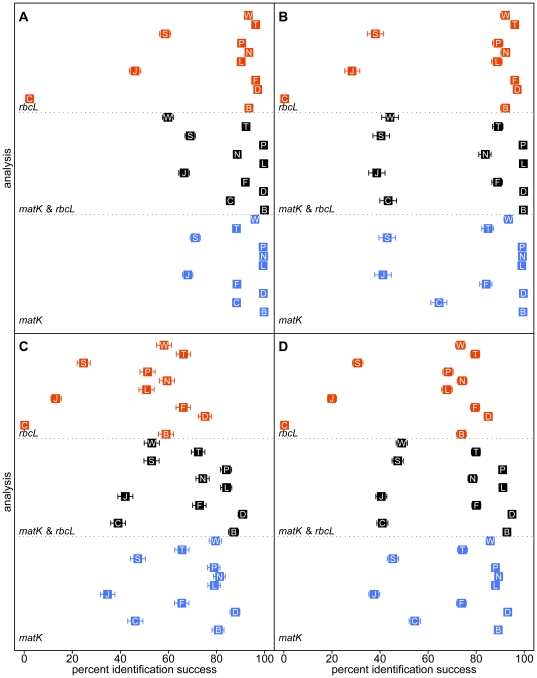
Tests of identification using full–length queries. Frequency of success, with 95% confidence intervals, for tests of (A) genus–level identification; (B) weak tests of species–level identification (i.e. those for which no congeners are represented in the data set); (C) strong tests of species–level identification (i.e. those for which congeners are represented in the data set); and (D) all tests of species–level identification. B = BRONX; C = CAOS; D = DNA–BAR/degenbar; F = forced (constrained) tree–search; J = SAP neighbor joining; L = pairwise matching (local alignment); N = NCBI-BLAST; P = pairwise matching (global alignment); S = SAP Barcoder; T = *de novo* tree–search; and W = WU-BLAST.

**Figure 3:**
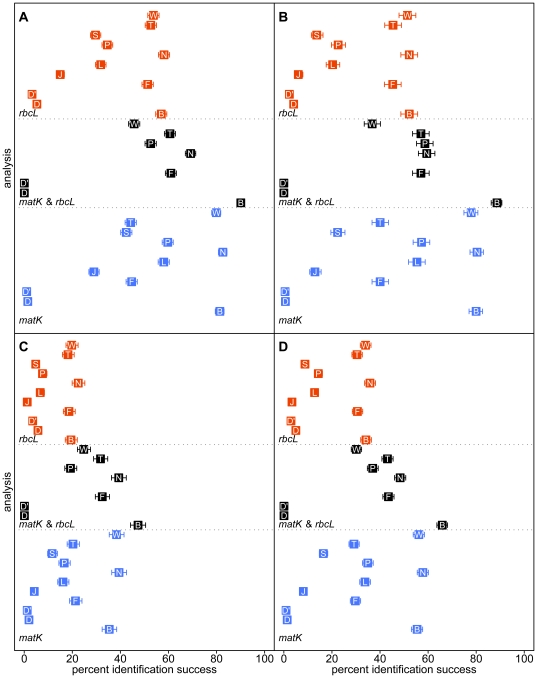
Tests of identification using mini–barcode queries. Frequency of success, with 95% confidence intervals for tests of (A) genus–level identification; (B) weak tests of species–level identification (i.e. those for which no congeners are represented in the data set); (C) strong tests of species–level identification (i.e. those for which congeners are represented in the data set); and (D) all tests of species–level identification. B = BRONX; D = DNA–BAR/degenbar with redundancy of 10; D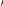 = DNA–BAR/degenbar with redundancy of 30; F = forced (constrained) tree–search; J = SAP neighbor joining; L = pairwise matching (local alignment); N = NCBI-BLAST; P = pairwise matching (global alignment); S = SAP Barcoder; T = *de novo* tree–search; and W = WU-BLAST.

In general, SIDEs had greater rates of success for ‘weak’ tests of species–level identification (i.e. those for which no congener was included in the dataset; Figs. 2B and 3B) than they had for ‘strong’ tests (i.e. those for which congeners are represented in the data set; Figs. 2C and 3C). Exceptions to this generalization include: WU-BLAST and both tree–building algorithms of SAP using full–length queries on the combined dataset; SAP Barcoder using full–length queries on the *matK* dataset; and DNA-BAR/degenbar using mini–barcode queries, The failure of WU-BLAST was inconsistent and unexpected (see below).

Among weak tests, no SIDE was able to correctly identify all queries–indicating that some of the tests that had been classified as weak, based on taxonomy, were in fact more challenging.

In general strong tests of species–level identification appear to be a much better means of discriminating among SIDEs than weak tests (compare Figs. 2B and 2C). Due to a high degree of congruence between weak and strong tests, weak tests do not distort the interpretation of trends in the overall results.

### Relative marker performance

In general *matK* data were better able to distinguish between genera and species than *rbcL* data–this disparity is well documented [25], [34], [44], [49], [50], [59]. Deviation from this general pattern can best be explained by difficulties with sequence alignment–*rbcL* is much simpler to align than *matK* (see below).

### Genus–level identification

Accurate genus–level identification is important for poorly described (or sampled) groups as well as for the enforcement of trafficking regulations. Regulators often list genera rather than attempting to maintain an exhaustive list of species for poorly described groups (e.g. *Encephalartos*, a CITES appendix 1 genus of cycad [109]).

Genus–level tests of identification were largely successful (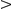
99%) for BRONX, DNA–BAR/degenbar, NCBI-BLAST, and pairwise matching when full–length *matK* data were used (Fig. 2A). It appears that *rbcL* is not variable enough to consistently distinguish among genera (the greatest success rate was 97%).

Species–level identification success is a good, but imperfect, predictor of genus–level identification success. For example, although DNA-BAR/degenbar performed best for species–level identification using full–length queries, BRONX was significantly better at genus–level queries in the same analysis (Table 1)–this is largely due to the explicit use of shared similarity in BRONX.

**Table 1:** Multiple comparison tests of SIDE genus– and species–level identification performance (*p* = 0.05).

comparison	genus–level tests			species–level tests		
	full–length	mini–barcode	overall	full–length	mini–barcode	overall
B vs. C	B 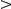 C	–	–	B 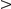 C	–	–
B vs. D	B 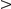 D	B 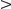 D	B 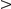 D	B 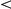 D	B 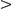 D	B 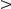 D
B vs. D 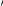	–	B 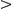 D 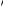	–	–	B 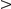 D 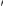	–
B vs. F	B 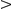 F	B 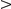 F	B 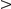 F	B 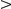 F	B 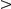 F	B 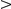 F
B vs. J	B 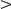 J	–	–	B 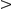 J	–	–
B vs. L	B 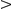 L	–	–	B 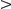 L	–	–
B vs. N	B 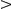 N	B 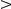 N	B 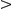 N	B 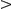 N	B 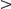 N	B 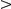 N
B vs. P	B 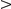 P	B 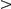 P	B 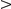 P	B 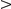 P	B 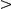 P	B 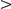 P
B vs. S	B 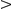 S	–	–	B 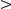 S	–	–
B vs. T	B 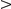 T	B 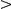 T	B 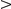 T	B 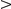 T	B 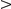 T	B 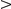 T
B vs. W	B 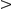 W	B 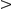 W	B 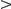 W	B 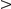 W	B 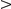 W	B 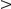 W
C vs. D	C 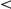 D	–	–	C 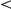 D	–	–
C vs. D 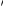	–	–	–	–	–	–
C vs. F	C 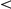 F	–	–	C 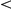 F	–	–
C vs. J	C = J	–	–	C = J	–	–
C vs. L	C 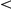 L	–	–	C 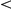 L	–	–
C vs. N	C 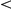 N	–	–	C 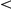 N	–	–
C vs. P	C 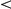 P	–	–	C 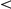 P	–	–
C vs. S	C 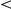 S	–	–	C 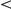 S	–	–
C vs. T	C 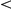 T	–	–	C 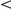 T	–	–
C vs. W	C 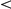 W	–	–	C 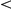 W	–	–
D vs. D 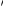	–	D 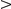 D 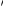	–	–	D 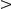 D 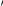	–
D vs. F	D 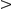 F	D 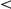 F	D 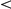 F	D 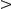 F	D 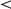 F	D 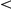 F
D vs. J	D 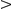 J	–	–	D 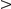 J	–	–
D vs. L	D 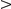 L	–	–	D 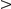 L	–	–
D vs. N	D 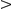 N	D 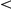 N	D 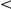 N	D 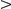 N	D 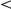 N	D 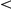 N
D vs. P	D 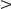 P	D 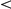 P	D 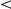 P	D 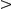 P	D 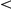 P	D 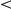 P
D vs. S	D 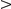 S	–	–	D 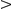 S	–	–
D vs. T	D 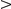 T	D 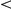 T	D 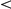 T	D 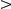 T	D 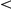 T	D 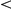 T
D vs. W	D 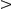 W	D 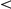 W	D 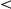 W	D 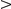 W	D 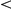 W	D 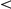 W
D 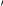 vs. F	–	D 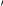 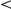 F	–	–	D 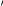 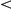 F	–
D 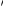 vs. J	–	–	–	–	–	–
D 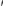 vs. L	–	–	–	–	–	–
D 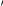 vs. N	–	D 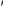 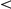 N	–	–	D 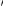 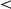 N	–
D 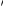 vs. P	–	D 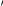 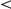 P	–	–	D 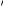 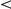 P	–
D 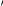 vs. S	–	–	–	–	–	–
D 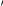 vs. T	–	D 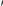 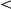 T	–	–	D 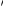 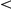 T	–
D 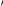 vs. W	–	D 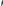 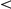 W	–	–	D 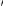 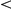 W	–
F vs. J	F 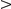 J	–	–	F 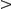 J	–	–
F vs. L	F 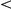 L	–	–	F 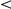 L	–	
F vs. N	F 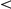 N	F 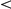 N	F 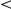 N	F 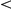 N	F 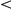 N	F 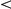 N
F vs. P	F 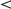 P	F 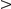 P	F = P	F 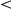 P	F 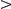 P	F = P
F vs. S	F 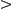 S	–	–	F 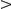 S	–	–
F vs. T	F = T	F = T	F = T	F = T	F = T	F = T
F vs. W	F 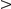 W	F 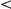 W	F = W	F 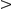 W	F 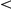 W	F = W
J vs. L	J 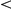 L	–	–	J 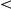 L	–	–
J vs. N	J 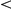 N	–	–	J 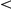 N	–	–
J vs. P	J 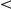 P	–	–	J 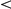 P	–	–
J vs. S	J 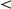 S	–	–	J 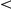 S	–	–
J vs. T	J 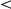 T	–	–	J 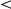 T	–	–
J vs. W	J 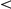 W	–	–	J 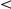 W	–	–
L vs. N	L 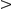 N	–	–	L = N	–	–
L vs. P	L = P	–	–	L = P	–	–
L vs. S	L 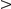 S	–	–	L 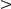 S	–	–
L vs. T	L 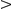 T	–	–	L 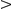 T	–	–
L vs. W	L 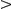 W	–	–	L 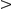 W	–	–
N vs. P	N 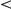 P	N 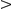 P	N 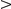 P	N = P	N 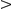 P	N 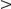 P
N vs. S	N 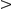 S	–	–	N 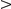 S	–	–
N vs. T	N 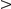 T	N 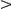 T	N 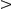 T	N 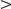 T	N 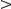 T	N 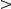 T
N vs. W	N 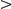 W	N 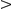 W	N 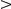 W	N 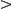 W	N 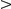 W	N 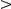 W
P vs. S	P 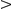 S	–	–	P 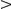 S	–	–
P vs. T	P 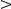 T	P 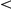 T	P = T	P 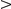 T	P 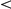 T	P = T
P vs. W	P 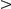 W	P 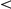 W	P = W	P 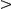 W	P 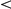 W	P = W
S vs. T	S 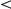 T	–	–	S 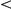 T	–	–
S vs. W	S 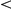 W	–	–	S 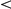 W	–	–
T vs. W	T 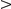 W	T 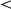 W	T = W	T 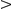 W	T 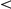 W	T = W

For identification of queries to genus, BRONX should be preferred over other SIDEs tested here.

### Mini–barcodes

Relative to full–length queries, identification success was much lower for mini–barcode queries (Fig. 3). Among the strong tests of species–level identification, the best score was 47%, achieved by BRONX with combined *matK* and *rbcL* data. This does not compare favorably to the best score achieved using full–length queries (91%, DNA–BAR/degenbar).

With the exception of DNA–BAR/degenbar, relative performance was similar among most SIDEs when mini–barcode queries were used (Table 1). Given the extremely poor species–level performance with mini–barcode queries and the corresponding low success in genus–level identification with single marker data (maximum of 82%), the strong synergistic performance of BRONX with combined queries is notable (90%; B in Fig. 3A).

From the data presented here, it appears that users of mini–barcodes should not expect accurate identifications even with the best available SIDE. It seems that accurate identification is not possible because there is not enough information in the mini–barcodes tested here.

### Relative SIDE performance

Statistically significant differential SIDE performance (Table 1) resulted in a range of interrater agreement values (Table 2). There is moderate agreement among SIDEs for full–length queries (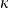 = 0.487–0.633). In contrast, little agreement can be detected among SIDES when mini–barcode queries are used (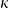 = 0.137–0.198). The lack of agreement is the result of conflicting sets of incorrect identifications combined with the high frequency of ambiguous identifications produced by mini–barcode queries. Analysis of combined markers produced slightly more agreement among SIDES when mini–barcode queries were used, whereas full–length queries produced a result in between the single marker results.

**Table 2:** Similarity of SIDE performance measured by Fleiss' index of interrater agreement (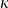).

	full–length queries	mini–barcode queries
*matK*	0.633	0.191
*matK* & *rbcL*	0.563	0.198
*rbcL*	0.487	0.137

Identification success did not consistently increase with combined data (Figs. 2, 3). Given that *matK* and *rbcL* are part of the same locus (plastid genome) and therefore track the same history [110] their combination should either increase identification success or have no observable effect. For BRONX, a synergistic effect was always observed when markers were combined. Simple pairwise matching displayed synergism except when genera were identified using mini–barcode queries. Synergism was generally, but not consistently, observed in tree–based methods (parsimony forced and *de novo* tree–search; SAP neighbor joining; and SAP Barcoder). A synergistic effect was also observed for DNA–BAR/degenbar when full–length queries were used, but slight antagonism was observed when mini–barcode queries were used. WU-BLAST, and to a lesser extent NCBI-BLAST, displayed an antagonistic effect when data were combined (see below).

### Simple pairwise matching

The type of alignment–local versus global–did not appreciably change the performance of simple pairwise matching (Table 1; Figs. 2, 3). The vast majority of differences in alignment occurred among pairs of highly dissimilar sequences. There were few changes in alignment among pairs of similar sequences–as a result the rate of identification success barely changed (i.e. both alignment algorithms were able to correctly recognize pairs of identical sequences).

For full–length queries, pairwise matching performed better, relative to other SIDEs, among strong tests of species–level identification where alignments were difficult (e.g. tree–search with *matK*), but worse when alignments were uncomplicated (e.g. most *rbcL* only analyses; L and P in Fig. 2C). The performance of pairwise matching was surpassed only by BRONX and DNA-BAR/degenbar (Table 1). For strong tests of species–level identification using mini–barcode queries, many algorithms consistently performed better than pairwise matching–only DNA–BAR/degenbar and both tree–building algorithms of SAP performed worse (Fig. 3C).

The performance of the NCBI implementation of BLAST was indistinguishable from pairwise matching in the statistical analysis of species–level identification, but in some circumstances (e.g. combined data, mini–barcode queries) is statistically more robust. Therefore, NCBI BLAST should be used in preference to pairwise matching.

Pairwise matching is not computationally efficient, but it is a consistent means of identifying query sequences. The success rate of pairwise matching is a useful performance threshold: worse performance is indicative of problems with a given SIDE; better performance indicates that the limits imposed by the pairwise alignment used in the matching algorithm (see Methods) have been overcome and/or additional variation (e.g. indels) has been extracted from the sequences. Thus, for the datasets examined here, species–level performance with full–length queries lower than 88% for *matK*, 68% for *rbcL*, and 91% for combined data are cause for concern as are species–level performance with mini–barcode queries lower than 35% for *matK*, 14% for *rbcL*, and 37% for combined data.

### Tree–based identification

The alignment of *matK* was complex–a median unaligned length of 1239 bp (IQR = 1080–1366 bp) became 4005 aligned positions. Of the aligned positions, 2187 were parsimony informative (54.6%). In addition, there were 778 informative indels for a total of 2965 informative characters. Portions of the MUSCLE alignment appear arbitrary and capricious.

The alignment of *rbcL* was uncomplicated–a median unaligned length of 624 bp (IQR = 612–633 bp) became 674 aligned positions. The majority of length variation was introduced by an *Epifagus virginiana* (L.) W.P.C.Barton sequence. The plastid genome of *E. virginiana* is greatly reduced presumably due its loss of photosynthetic function–many gene regions are highly modified and/or apparently non–functional [111]. Of the aligned positions, 388 were parsimony informative (57.6%). In addition, there was an informative indel.

Despite the greater number of parsimony informative positions in the *matK* matrix, there was no significant difference in tests of species–level identification between the two markers (T in Fig. 2).

As indicated by the performance of the simple pairwise matching algorithm, the ambiguity of the *matK* alignment is likely responsible for inconsistent tree–based performance–it is difficult for an alignment program to exactly mirror the arbitrary and capricious alignment of reference sequences when adding query sequences. As a result the placement of the query sequences in the phylogenetic tree may deviate from the reference sequences they most resemble [11], [22], [23].

The most parsimonious trees were 62,315 steps (CI = 0.10, RI = 0.85) for the *matK* dataset; 8146 steps (CI = 0.09, RI = 0.89) for the *rbcL* dataset; and 71,459 steps (CI = 0.10, RI = 0.86) for the combined datasets (all tree statistics were calculated excluding uninformative characters). Using the strict consensus of these trees as positive constraints, the forced tree–search was statistically indistinguishable from the *de novo* tree–search (F and T in Fig. 2; Table 1). The computer time required for the forced tree–search is however greatly reduced due to the restricted portion of tree–space examined.

The CAOS algorithm did not perform well (C in Fig. 2)–all other SIDEs were significantly better (Table 1). This is probably best explained by the rampant homoplasy in both datasets (ensemble CI = 0.09, 0.10 [112]). CAOS seeks ‘pure’ and ‘private’ attributes to be used for query classification–pure attributes cannot be homoplastic and private attributes usually are not [it is possible for a private attribute to be homoplastic if the other occurrence(s) do not define clades]. In either case, homoplastic characters greatly reduce the number of classifiers that CAOS can use and thereby reduce the performance of the CAOS algorithm. Irregardless of homoplasy, the CAOS algorithm is dependent upon tree topology and therefore benefits from, and is limited by, the method that was used to build the CAOS reference tree.

Parsimony–based tree–building methods consistently produced more correct species–level identifications than either of the SAP [13], [20] tree–building algorithms (F and T vs. J and S in Fig. 2; Table 1). It appears that sequence alignment plays a role in the differential performance between parsimony and SAP–the parsimony methods align all reference sequences with MUSCLE whereas SAP aligns a subset of the reference sequences using ClustalW2. Even when alignment is unambiguous (i.e. *rbcL*) the performance is not equal (parsimony is superior). Thus both the method of tree construction and tree interpretation are responsible for performance differences. SAP's Barcoder algorithm is much more computationally intensive than the neighbor joining algorithm, but it significantly out performed the neighbor joining algorithm and therefore should be used preferentially (J and S in Fig. 2; Table 1).

Relative performance rankings using mini–barcode queries were similar to full–length queries (Fig. 3; Table 1).

Unlike other SIDEs, all tree–based methods are forced to assume that the identified terminals are ‘monophyletic’ [11]. The frequent violation of this assumption [113], [114] lowers the performance of all tree–based SIDEs. The impact of terminal non–monophyly on the data presented here is not known.

As previously noted [11], [115], when alignment is not a concern, conventional tree–based methods seem to offer a mediocre, but viable, means of identification (e.g. *rbcL*), but when alignment is difficult, tree–based methods should be avoided (e.g. *matK*) with preference given to BRONX, DNA-BAR/degenbar, NCBI-BLAST, and pairwise matching.

### DNA–BAR/degenbar

For strong tests of species–level identification using full-length queries DNA–BAR/degenbar was significantly better than all other SIDEs (D in Fig. 2; Table 1). However, DNA–BAR/degenbar failed to correctly identify almost all mini–barcode queries (maximum 11.24% success). Tripling the coverage (redundancy) of the reference database produced significantly worse results (D and D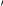 in Fig. 3; Table 1). The failure of DNA-BAR/degenbar with mini–barcodes can be traced to the scoring algorithm's use of logical exclusions (i.e. *x* NOT *y*) [11]. DNA-BAR/degenbar fails because absence of evidence (i.e. a short query sequence) is taken as evidence of absence.

DNA–BAR/degenbar is highly effective when there is little missing data (e.g. full–length queries), but this SIDE should not be used when query length differs substantially from reference sequence length (e.g. mini–barcode queries). This failing results in the placement of DNA–BAR/degenbar below all other methods in the overall rankings (Table 1).

### BLAST

The performance of BLAST implementations on single marker datasets was not very different from one another, but the NCBI implementation was significantly better than the WU implementation (Table 1). In either case, the performance was not outstanding (N and W in Fig. 2).

The utter failure of WU-BLAST with combined *matK* and *rbcL* queries was therefore unexpected. The method of calculating a unified BLAST score for combined *matK* and *rbcL* queries (see Methods) cannot be solely responsible for this failure because the same method was used for both BLAST implementations and the NCBI implementation performed as expected (i.e. midway between its performance for strong tests of species–level identification using single marker queries). The calculation of unified scores for the combined dataset could however be improved as evidenced by the better performance of the simple pairwise matching algorithm.

Both BLAST implementations maintained their relative ranking when confronted with mini–barcode queries.

Past comparisons of barcode SIDEs [11] have found BLAST performance to be stronger than other procedures. In relative terms, the performance reported here is not as good–likely due to more stringent criteria for judging identification success (see Methods). BLAST is a rapid means of query sequence identification, but other SIDEs provide greater accuracy and consistency. If BLAST is used, the NCBI implementation should be preferred.

### BRONX

For genus–level identification, BRONX was consistently superior to other SIDEs tested here (Figs. 2A, 3A; Table 1). For species–level identification, BRONX consistently outranked all other SIDEs save DNA-BAR/degenbar (Figs. 2C, 3C; Table 1). The failure of DNA-BAR/degenbar with mini–barcode queries served to increase the overall rank of BRONX above that of all other SIDEs.

The use of logical exclusions in the DNA-BAR/degenbar scoring algorithm [11], but not in the BRONX scoring algorithm explains the superior performance of DNA-BAR/degenbar in tests where the length of the queries closely matches the length of the sequences in the reference database. The disadvantage of using logical exclusions is made abundantly clear when using mini–barcode queries–DNA-BAR/degenbar reliably and catastrophically fails. For this reason BRONX was explicitly designed to use only unambiguous context/text presence in its scoring. Unfortunately this decreases the performance when query sequence length closely matches that of the reference database.

BRONX should be used in preference to other SIDEs when there is imperfect overlap between query and reference sequences (e.g. mini–barcode queries against a full–length database) or when identifications to genus are desired.

### Conclusions

SIDEs that do not consistently perform as well as pairwise matching are manifestly flawed. Thus, the data presented here suggest that due to inconstant performance no tree–based method should be used for barcode sequence identification.

The performance of pairwise matching was better than WU-BLAST, but not statistically distinguishable from that of NCBI-BLAST. Given that NCBI-BLAST is computationally much faster than pairwise matching, NCBI-BLAST should be used in preference to pairwise matching.

BRONX performs better than all other SIDEs when there is imperfect overlap between query and reference sequences, but when the query sequence length closely matches the reference database, DNA-BAR/degenbar exhibits superior performance. BRONX consistently produced better identifications at the genus–level.

## Supporting Information

Dataset S1 **A comma separated text file containing: genus, specific epithet, specimen identification number used in this study,** ***matK*** **GenBank accession,** ***matK*** **DNA sequence,** ***matK*** **mini–barcode,** ***rbcL*** **GenBank accession,** ***rbcL*** **sequence,** ***rbcL*** **mini–barcode, and an indication of use in the reference dataset.**
(CSV)

Dataset S2 **A FASTA formatted text file of** ***matK*** **sequences aligned with MUSCLE (used for some tree–based identifications).** Sequence names correspond to the specimen identification number in Dataset S1.
(FASTA)

Dataset S3 **A FASTA formatted text file of** ***rbcL*** **sequences aligned with MUSCLE (used for some tree–based identifications).** Sequence names correspond to the specimen identification number in Dataset S1.
(FASTA)
